# Visual Acuity Among Portuguese School-Aged Population

**DOI:** 10.3390/jcm14082824

**Published:** 2025-04-19

**Authors:** María Ibeth Peñaloza-Barbosa, Clara Martinez-Perez, Cristina Andreu-Vázquez, Miguel Ángel Sánchez-Tena, Cristina Alvarez-Peregrina

**Affiliations:** 1 School for Doctoral Studies and Research, Universidad Europea de Madrid, 28670 Madrid, Spain; mariaibeth2008@gmail.com; 2 Instituto Superior de Educação e Ciências de Lisboa (ISEC Lisboa), Alameda das Linhas de Torres, 179, 1750-142 Lisboa, Portugal; masancheztena@ucm.es; 3 Department of Veterinary, Faculty of Biomedical Science and Health, Universidad Europea de Madrid, 28670 Madrid, Spain; cristina.andreu@universidadeuropea.es; 4 Department of Optometry and Vision, Faculty of Optics and Optometry, Universidad Complutense de Madrid, 28037 Madrid, Spain; cristina_alvarez@ucm.es

**Keywords:** children, refractive errors, amblyopia, early detection

## Abstract

**Background/Objectives:** Visual acuity (VA) screening is highly valid in school-aged children for detecting visual impairments. This study aims to provide a comprehensive profile of VA among school-aged children (6–17 years old) from Lisbon, Portugal. Specifically, it focuses on estimating the prevalence of abnormal VA (VA < 0.8 in the worst eye) and establishing age-specific normative reference values (percentiles) for monocular and binocular VA within this urban sample. **Methods:** We performed an observational, descriptive, and cross-sectional study that included healthy children aged 6 to 17 years. Convenience sampling was used in three educational centers in Lisbon. Retinoscopy and monocular VA (right eye, left eye, and the eye with the lowest) and binocular VA were assessed using the Herman Snellen optotype. Descriptive statistics, prevalence of abnormal VA (worst-eye VA < 0.8), and monocular and binocular VA percentiles were calculated for the whole cohort and by age. **Results:** The sample consisted of 2215 children (49.6% of males; and 9.4 ± 2.6 years old). Binocular VA, right-eye VA, left-eye VA, and VA worst eye in the study cohort showed ranges of 0.92–1.00, 0.90–0.95, 0.89–0.97, and 0.85–0.94, respectively, for ages 6–17 years. In total, 86.8% of the study population had a normal VA (≥0.8). VA deficit was mild (0.8 < AV ≥ 0.3), moderate (0.3 < AV ≥ 0.125), and severe (VA < 0.125) in 10.5%, 1.1%, and 1.6% of children, respectively. **Conclusions:** Our study provides both age-specific normative reference values for visual acuity in Portuguese children and prevalence estimates for visual acuity deficits. These findings offer critical data to support early detection strategies and the development of age-adjusted vision-screening protocols in school-aged populations.

## 1. Introduction

Visual acuity (VA) capability, referring to the eye’s faculty to discriminate shapes and details of objects at a specific distance, is one of the fundamental elements of visual functions [1,2,3]. The development of this visual function begins at birth, followed by the progressive physiological development of the retina and the optic pathway throughout childhood [1]. Proper development of visual function during childhood is crucial for binocular vision, acquisition of visual information, and the development of intelligence [4]. Thus, abnormal visual development in early life can hinder sensory and cognitive growth and raise the risk of irreversible visual disabilities, leading to long-term negative effects [5].

The accurate measurement of VA is one of the most challenging issues in the pediatric population for primary-care professionals [6]. The measurement of uncorrected VA typically represents the first clinical step in detecting potential visual anomalies [7,8]. The existing method of choice for screening VA in children aged from 3 to 5 years is taken with standardized optotypes, according to the principle of Bailey and Lovie [9]. However, non-standardized optotypes continue to be used in clinical practice. Furthermore, there is no consensus on the criteria for measuring VA in the pediatric population, as such criteria vary between countries and even among medical institutions within the same country [10,11]. Therefore, to improve the detection process of visual system disorders, standardization of VA assessment methodology is required, as well as the development of percentile curves that reflect the progression of such disorders based on the individual’s age.

With a prevalence of 1% to 5%, amblyopia, the disruption in the typical neuronal development of the immature visual system, is one of the leading causes of visual disability in children [12,13]. This condition negatively affects the child’s quality of life, so it is essential to address it between the ages of 5 and 7 for maximum efficacy [14,15]. Currently, the VA criteria used to refer suspected cases of amblyopia in children, due to refractive errors, are not consistent at an international level, especially in preschool-aged children [16]. Early diagnosis of factors impairing vision development is crucial for the development and preservation of good VA, as early treatment allows for the reversal of cortical damage [17]. Nevertheless, exams to identify amblyopia, including VA measurement, are a non-invasive and simple procedure to develop, and may be a feasible option for children and their parents [10,18]. It is important to detect possible visual impairments, such as myopia, at early stages of development in order to reduce the risk of suffering irreversible loss of VA.

To distinguish normal from abnormal VA, it is essential to establish criteria of VA value ranges representative of normality. According to Snellen and Landolt [19], normal VA is defined as 1.0 (logMAR). However, it is known that this parameter can adopt values higher than 1.0 [19,20]. Considering that the development of the visual system varies throughout life and according to environmental factors, the normality criteria for VA should be established according to the individual’s age [21,22]. The VA value of 1.0, within the field of ophthalmology and in visual screening, seems not to be appropriate as a referral criterion for all ages, and further investigation is required to reach a consensus within the scientific community.

Currently, the lack of a standardized method that accurately assesses VA causes concern among the population. This is because the referral of children to an ophthalmologist due to low VA varies considerably depending on the medical institution, the professional involved, and the applied test [10]. Considering that VA screening is highly valid during the school years for detecting visual deficiencies, the present work aims to provide a comprehensive information set on the prevalence of abnormal VA and its deficit in a Portuguese school-aged population between 6 and 17 years old.

This study addresses a critical gap in national data by establishing age-specific reference values for monocular and binocular VA in Portuguese children. The absence of such normative benchmarks limits the capacity for early detection and timely intervention in cases of refractive errors or amblyopia. By identifying age-related patterns and quantifying the extent of visual deficits, this research provides robust evidence to support the development of standardized screening strategies within school health programs and pediatric care. These results also offer a practical tool for researchers and clinicians to better interpret VA outcomes in the local pediatric population and to align referral criteria with population-specific norms.

## 2. Materials and Methods

### 2.1. Study Design

This is an observational, descriptive, and cross-sectional study. This research complied with the principles of the Helsinki Declaration and was approved by the Ethics Committee of the Higher Institute of Education and Sciences (ISEC) of Lisbon (Portugal) (CE/2022/03/01). Parents or legal guardians of the participants signed an informed consent document.

### 2.2. Study Population

Between the months of July and September of the years 2021, 2022, and 2023, seven schools in the city of Lisbon were contacted via letter to invite them to participate in the study. Parents of children from Agrupamento de Escolas Póvoa De Santa Iria, Agrupamento de Escolas de Alcabideche, and Colégio Campo de Flores who agreed to participate received information about the study and signed an informed consent form. The inclusion criterion was ages from 6 to 17 years. Children with any ocular and/or systemic disease reported by their parents were excluded from the study. The subjects were examined during the academic years 2021–2022, 2022–2023, and 2023–2024. Each child was assessed only once, with no repeated evaluations across the years. The same age range (6 to 17 years) was included in each academic year, depending on the age distribution at each participating school.

### 2.3. Materials

The VA was measured using the Herman Snellen optotype [19]. The Snellen chart represents the minimum recognizable separation between two objects at a determined distance. Following the guidelines of the American Optometric Association, the test was conducted using a chart rather than a projector, placed at three meters of distance from the subjects, as a reliable method to determine VA in children [23]. The optotypes used were eye charts with visual symbols, such as letters, numbers, and figures of different sizes. The test was performed under photopic conditions. In the standardized optotypes used, the rows have the same number of letters or symbols, and the space between rows and letters is proportional to the size of these letters. This method is highly precise and reliable, and its performance requires adequate spatial orientation, which develops by the age of 5 [19].

### 2.4. Procedure

For the collection of census data and the measurement of VA, the monocular and binocular decimal measures of each individual were included. Qualified professionals performed screening at the three selected schools. Each school prepared an appropriate and well-equipped room for the test, where subjects were organized by age and school grade. The designated room had two separate areas: one to place the whole group, and another one where various tests were conducted. In spite of children attending in groups, the test was conducted individually. In this way, children could not interact with each other, thus avoiding potential bias in data collection.

Distance VA was measured with and without optical correction, including the use of glasses or contact lenses, and recorded binocularly (VA Binocular), as well as monocularly, for the right eye (VARE), left eye (VALE), and the eye with the lowest VA (VA worst eye). As part of the visual screening, retinoscopy was performed after the VA assessment to estimate refractive error without determining the final corrective prescription. Directional E and mixed Snellen optotypes were placed at eye level at a distance of three meters. An occluder was used for children over 7 years old, while a patch was used for those under 7. Directional E optotypes were used for children under 7 years old, and letter optotypes were used for those over 7.

To establish norms for VA, it is crucial to consider that, at the 6/6 scale, a person can visualize an image that encompasses an angle of 5 min on their retina when it is at a distance of 6 m. This represents the Minimum Angle of Resolution (MAR) and denotes optimal vision. Expressed as a decimal, this value is 1. The MAR is calculated as 1/1 = 1 and log10 = 0, which means that in logMAR notation, normal VA is 1 [23]. Visual Acuity Rating (VAR) was determined by counting the number of letters correctly identified and calculating the log MAR according to the following formula: (85—letters correct) × 0.02, or using the formula VAR = 100–50 × logMAR, where a value of 100 corresponds to a VA of 20/20 [19,24].

Following the WHO (World Health Organization), the definition of the various visual acuities was normal VA of worst eye (VA ≥ 0.8) and abnormal VA of worst eye (VA < 0.8). The degree of VA deficit was defined as mild (0.8 < AV ≥ 0.3), moderate (0.3 < AV ≥ 0.125), and severe (VA < 0.125) [25].

### 2.5. Statistical Analysis

Descriptive statistics were carried out to summarize the characteristics of the children included in the study, as well as their values of VARE, VALE, and VA worst eye. Continuous variables were presented as means ± standard deviations (SDs) when they followed a normal distribution, or medians and interquartile ranges [Q1, Q3] if the variable did not follow a normal distribution, as determined by a Shapiro–Wilk test. Categorical variables were presented as absolute and relative frequencies. Additionally, percentiles (p5, p10, p25, p50, p75, p90, and p95) of VA for the right eye, left eye, worst eye, and binocular VA were calculated for the entire cohort and by age following the Bueno-Matilla vision unit [26]. This unit includes reference percentile curves according to the age of the children. It was calculated that a minimum of 30 children per age was necessary to describe VA accurately (standard deviation of VA, 0.15–0.19; precision error, 0.05–0.07; and confidence level, 95%).

The prevalence of abnormal VA (VA in the worst eye < 0.8) and its 95% confidence interval (CI 95%) were calculated using the Wilson method for the entire cohort and by age group (6–17 years old). The degree of VA impairment (mild, VA between 0.3 and 0.8; moderate, VA between 0.125 and 0.3; severe, VA < 0.125) was also determined. To determine the possible association between the prevalence of abnormal VAs and age, ages were grouped into three-year intervals (6–8, 9–11, 12–14, and 15–17), and chi-square tests, adjusted residuals, and logistic regression analyses were conducted. Binocular VA and VARE, VALE, and VA worst eye were also described for children with normal and abnormal VA (including mild, moderate, and severe VA impairment). All analyses were conducted using the STATA v.17 software package (StataCorp LLC, College Station, TX, USA).

## 3. Results

Vision screenings were conducted on a total of 2215 children aged 6 to 17 years. Among them, 51 children (2.3%) were excluded from the study due to missing data or errors in VARE and/or VALE and/or binocular VA records.

Table 1 summarizes the gender and age distribution of the 2164 children included in the study. The proportion of boys and girls was 49.6% and 50.4%, respectively. The mean age for boys was 9.4 ± 2.5 years, and that of the girls was 9.3 ± 2.6 years.

Table 2 describes monocular VA (VARE, VALE, and VA worst eye) and binocular VA for each age group. Monocular VA in the overall cohort of children included in the study was 0.92 ± 0.17 for the right eye, and 0.92 ± 0.18 for the left eye. VA in the worst eye was 0.90 ± 0.19. Binocular VA in the cohort of 2164 children was 0.95 ± 0.15 (Table 2). Percentiles of binocular VA and VARE, VALE, and VA worst eye for each age group are shown in Table A1.

Table 3 shows the prevalence of abnormal VA and the degree of VA impairment by age. In 286 children (13.2%, 95% CI: 11.9% to 14.7%), VA in the worst eye was lower than 0.8. VA impairment was mild (i.e., worst-eye VA between 0.3 and 0.8) in 228 children (10.5%), moderate (i.e., worst-eye VA between 0.125 and 0.3) in 23 children (1.1%), and severe (i.e., worst-eye VA < 0.125) in 35 children (1.6%). Children aged 9–11 and 15–17 years have significantly higher odds of having abnormal VA compared to those aged 6–8 years (OR = 1.81, *p* < 0.001; and OR = 1.74, *p* = 0.042, respectively). Specifically, the prevalence of abnormal VA for these age groups is 16.3% and 15.7%, respectively, versus 9.6% for the 6–8 age group.

**Figure 1:**
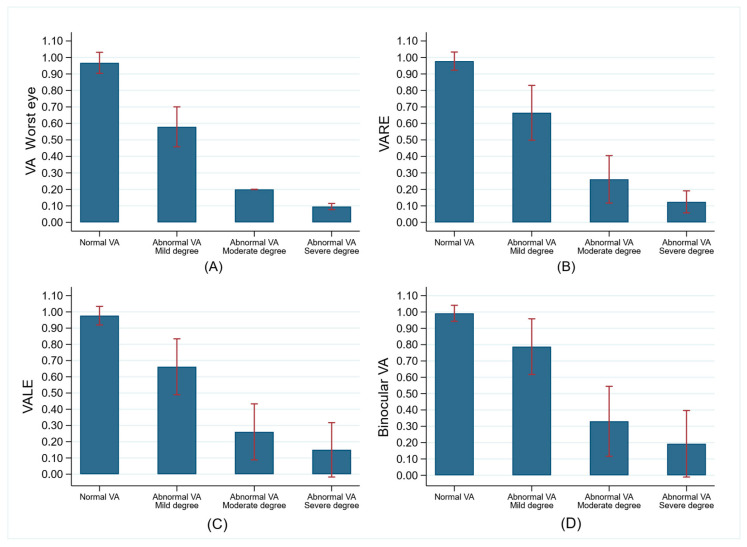
Comparison of monocular and binocular visual acuity (VA, mean and SD, and Snellen decimal notation) between children with normal (VA in worst eye > = 0.8, n = 1878) and abnormal VA (VA in worst eye < 0.8; n = 286). (**A**) Comparison of worst-eye VA between children with normal VA and those with mild, moderate, and severe VA impairment. (**B**) Comparison of VARE between children with normal VA and those with mild, moderate, and severe VA impairment. (**C**) Comparison of VALE between children with normal VA and those with mild, moderate, and severe VA impairment. (**D**) Comparison of binocular VA between children with normal VA and those with mild, moderate, and severe VA impairment.

Both binocular and monocular VA values for the RE, LE, and worst eye were significantly lower in children with mild, moderate, and severe VA impairment compared to children with normal VA (worst-eye VA ≥ 0.8; Figure 1).

## 4. Discussion

The present study provides valuable information on the prevalence of normal and abnormal VA, as a function of age, in a Portuguese school population aged 6–17 years. To the best of our knowledge, this is the first study in Portugal to carry out a visual screening in a wide age range, covering practically all school ages (6–17 years). In total, 86.78% of the study sample had optimal VA (worst-eye VA ≥ 0.8), and only 13.2% showed a VA deficit (worst eye, <0.8). Among the children with visual impairment, the majority (10.54%) had a mild degree of visual impairment (VA worst eye, 0.3–0.8).

There is currently no consensus on VA measurement as there are external factors that influence this procedure, including the type and method of optotype presentation [8,21,24]. While the use of a six-meter test distance is considered standard for measuring VA in adults, smaller working distances (1.5–3 m) allow for excellent repeatability and reliability for measuring VA in children [24]. In this study, following the guidelines of the American Optometric Association, the Snellen foil optotype at a distance of 3 m was used for all age groups as a reliable method to determine VA in children [19,24]. The test was easy for patients to interpret and was conducted by qualified personnel. Assessment of visual acuity in far vision was verified with the standardized optotype (symmetrical letters and directional E), using the decimal VA or logMAR as the measurement scale.

Based on the physiology of ocular development, it has been widely described that VA in children varies with age [1,18]. Assessment of VA in childhood (even during the first month of life) is key to maintaining good vision and preventing potential vision problems [10,27]. In our study, children were classified by age (6–17 years), to accurately assess monocular and binocular VA with best correction. The study population showed optimal VA (≥0.8), with VARE, VALE, and binocular VA ranges of 0.90–0.95, 0.89–0.97, and 0.92–1.00, respectively, for ages 6–17 years. The authors of a previous study carried out in a Spanish population of the same age range obtained an VARE, VALE, and binocular VA of 0.92 ± 0.17, 0.92 ± 0.18, and 0.95 ± 0.15, respectively [28]. Considering specific ages, in our study, 6-year-old children have a VARE and VALE of 0.95 in both cases, and a binocular VA of 0.97. Furthermore, 91.52% of children of this age have a normal VA (≥0.8). These results agree with previous studies conducted in Turkey, in which 94.5% of 6-year-old children had a VARE and VALE equal to or greater than 0.63, and of these, 86.6% had a VA of 1 [29]. The author of that study found that 86.3% of 6-year-old children had an optimal VA (equal to or greater than 0.6). Another study showed that 67% of the population of US 6-year-olds have a VARE equal to or greater than 1 [30]. Similarly, a study of a Hong Kong 6-year-old population indicated that 97.3% to 96.2% had a VARE > 0.5 [31]. Moreover, a Portuguese study found that 86.3% of 6-year-olds had a normal VA (equal to or greater than 0.6) [32]. However, these VA values, according to our study, would be categorized as abnormal, being equal to or lower than 0.8, which could explain the differences between the two studies. In addition, the methodology used was different in both cases: they employed a Lea Chart, and we used a Snellen Table [32]. These differences could be due to a lack of methodological standardization.

Regarding the 9-year age group, our study observed a mean VARE and VALE of 0.91, and a binocular VA of 0.94. Approximately 84% of children in this age group had normal VA, only 13% had abnormal mild VA (0.3–0.8), and only 2% had severe visual impairment (<0.125). However, the existing literature shows a lower prevalence of severe visual impairment than what we have found in our study. For example, a recent study found a prevalence of severe visual impairment of 0.14% (binocular VA ≤ 0.3) in a population of Iranian children aged 9 years on average [33]. This difference between the two studies may be because the children in the previously mentioned study had refractive error and amblyopia [33], while our sample did not.

Our study showed that 86.81% of children in the 12-year age group had normal VA. The mean VARE, VALE, and binocular VA found were 0.92, 0.91, and 0.94, respectively. These data are consistent with a study of members of the Chinese population aged 12–16 years that found normal VA in 97.47% of the subjects [34]. Moreover, the study developed in Australian children aged 10–12 years shown a VA demand of 0.47 [35]. However, these authors define normal VA as equal to or greater than 0.6, whereas in our study, it is 0.8, which may lead to an overestimation of VA values in these works [34,35].

In general, studies assessing VA in child populations do not distinguish between age groups (VA for each age, e.g., 6 years, 7 years, etc.), but they show VA values in age ranges (VA of different ages, e.g., 6–7 years, 8–9 years, etc.). For example, there was a study conducted with Chinese children aged 7–12 years [36]. The authors obtained a mean monocular VA in the range of 0.8–1 and stated that children aged 7–9 years and 10–12 years have a VA of 0.75 and 0.86, respectively. In our study, children of the same age have a higher normal VA than in this study (RE and LE ≥ 0.90); 84–90% of cases have a normal VA (≥0.8) [36]. On the other hand, research on a Chinese population aged 5–7 years reveals that 88% of the subjects have normal VA (>0.625) [37]. While, in our study, the normal VA for these ages is close to 92%, the average (i.e., abnormal) visual impairment is 8%. In this sense, the difference in VA values may be due to the greater complexity of oriental teaching conformed by intricate alphabetic scripts, which include precise characters and strokes. It is related to the logographic writing systems, such as Chinese or Japanese, which are more visually intricate compared to our Western systems. This could imply a greater demand when measuring VA in far vision in the Eastern populations.

According to the WHO, VA can be classified as normal and abnormal, and visual impairment as mild, moderate, or severe [25]. This classification allows for a thorough examination of vision in child populations and reduces the prevalence of uncorrected refractive error [25]. In our study, 13.22% of the population had abnormal VA (worst eye, <0.8), and of these, the largest proportion (10.54%) had mild visual impairment (VA 0.3–0.8). Only 1.62% of the population had a severe reduction in VA (<0.125). Similarly, previous studies conducted in populations of Nigerian and Ethiopian children aged 4–18 years found a prevalence of visual impairment (VA < 0.5) of 1.9% (4–15 years), 1.4% (4–18 years) and 5.8% (5–16 years), respectively [38,39,40]. Recent work found 1.8% and 2.8% moderate abnormal VA (<0.5) in a school population aged 5–15 years in Ethiopia and Australia, respectively [41,42]. Similarly, a study of a Nigerian population aged 7–16 years showed that only 1% of subjects have a VA deficit (<0.3) [43]. However, other studies suggest that the highest prevalence of abnormal vision is in child populations. For example, a Malaysian study of children aged 7–12 years found reduced VA (<0.5) in 41% of the population [44]. Other studies, such as the Chinese multiethnic study conducted in a population of children aged 5–16 years, found decreased VA (<1) in 11.4% [45]. These higher percentages of decreased VA, compared to our study, may be due to the sociodemographic differences between the study populations themselves and the fact that these studies include children with ocular pathologies [44]. In addition, the variability in the criteria used by the authors to define the limits of impairment VA (1.5–0.5) and the lack of description of the groups with reduced VA make it difficult to make a robust comparison. Our study follows the WHO classification, which precisely defines VA impairment according to three categories (mild, moderate, and severe) [25].

The findings of this study have direct implications for the development of school-based vision-screening programs in Portugal. By providing age-specific normative values for visual acuity and identifying age groups with a higher prevalence of visual deficits (particularly between 9 and 11 years old), this work supports the implementation of more targeted screening strategies and consistent referral criteria. Furthermore, the inclusion of percentile data allows professionals to interpret each child’s VA in relation to their age group, which may be useful in borderline cases or when longitudinal follow-up is needed. These percentiles complement traditional threshold-based approaches and can contribute to more nuanced and effective vision-screening protocols. In addition, as the study excluded children with known systemic or ocular diseases, the prevalence of abnormal visual acuity may have been slightly underestimated. This decision was made to focus the analysis on children without diagnosed conditions, aligning with the purpose of population-level school-based screenings. It is also important to note that while this study measured visual acuity and included retinoscopy, it did not fully explore the underlying causes of reduced VA. Future research should address this limitation by linking VA outcomes to detailed refractive, anatomical, and neurological findings.

One of the limitations of this study is the type of screening carried out: it is effective in assessing visual impairment (myopia, hyperopia, and astigmatism), but it is not useful in detecting ocular pathology. However, as the aim of this study is not ocular pathology, this type of screening does not imply a problem for the conclusions of the study. In addition, another limitation of the study is the unbalanced sample size in each age group, so there is a need for equal representation of all age groups. Finally, other anatomical, physiological, and optical factors, such as retinal eccentricity, refractive error, pupil size, luminance, and contrast, may affect VA acquisition [24]. These factors were not taken into account in our study, but they should be considered in future research.

Despite the limitations mentioned above, this is considered a preliminary study due to its use of convenience sampling and limited clinical scope (focused on visual acuity and retinoscopy), providing an initial basis for future studies in this area of research in Portugal. Another limitation of this study is the use of convenience sampling, which may introduce selection bias and limit the generalizability of the findings to the entire Portuguese school-aged population. Furthermore, it is a study that analyzes VA and retinoscopy with the intention of efficiently determining visual impairment in school-aged children. Based on these data, future work could explore percentile curves of refractive error and ocular biometry, allowing for a better understanding of how these parameters relate to visual acuity distribution and screening outcomes [31]. All of this will contribute to the generation of knowledge, the reduction of the incidence of visual impairment in the school population, and the improvement of the economic performance of the health sector. Our study establishes a starting point for the improvement of future public health policies and the investigation of underlying factors affecting visual health in a young Portuguese population. Although this study focuses on visual acuity as the main parameter to assess visual impairment, we acknowledge that this is only one dimension of a broader concept. Visual impairment can also include deficits in contrast sensitivity, visual field, or neurological processing. However, our aim was to provide standardized, population-based reference values for VA in school-aged children, which remains the most accessible and widely used indicator in vision screenings. Future studies may expand this work by incorporating additional measures to reflect the full complexity of visual function.

## 5. Conclusions

Our study provides both age-specific normative reference values for visual acuity in Portuguese children and prevalence estimates for visual acuity deficits. These findings offer critical data to support early detection strategies and the development of age-adjusted vision-screening protocols in school-aged populations.

## Figures and Tables

**Table 1:** Sociodemographic characteristics of the children included in the study.

	Total (n = 2164)	Boys (n = 1073; 49.6%)	Girls (n = 1091; 50.4%)
Age (mean ± SD)	9.35 ± 2.56	9.37 ± 2.50	9.32 ± 2.62
Age (n (%))			
6 years	283 (13.08)	138 (12.86)	145 (13.29)
7 years	298 (13.77)	135 (12.58)	163 (14.94)
8 years	321 (14.83)	163 (15.19)	158 (14.48)
9 years	320 (14.79)	156 (14.54)	164 (15.03)
10 years	336 (15.53)	171 (15.94)	165 (15.12)
11 years	279 (12.89)	145 (13.51)	134 (12.28)
12 years	91 (4.21)	55 (5.13)	36 (3.30)
13 years	60 (2.77)	31 (2.89)	29 (2.66)
14 years	55 (2.54)	26 (2.42)	29 (2.66)
15 years	42(1.94)	20 (1.86)	22 (2.02)
16 years	50 (2.31)	20 (1.86)	30 (2.75)
17 years	29 (1.34)	13 (1.21)	16 (1.47)

**Table 2:** Monocular (right eye, left eye, and worst eye) and binocular visual acuity (VA, Snellen decimal notation) in children of different ages.

	Monocular VA			
Age	VARE (Mean ± SD)	VALE (Mean ± SD)	VA Worst Eye * (Mean ± SD)	Binocular VA (Mean ± SD)
6 years	0.95 ± 0.11	0.95 ± 0.12	0.94 ± 0.13	0.97 ± 0.09
7 years	0.94 ± 0.13	0.95 ± 0.13	0.92 ± 0.15	0.97 ± 0.10
8 years	0.95 ± 0.14	0.93 ± 0.15	0.92 ± 0.16	0.96 ± 0.12
9 years	0.91 ± 0.20	0.91 ± 0.19	0.89 ±0.20	0.94 ± 0.17
10 years	0.90 ± 0.21	0.89 ± 0.22	0.88 ± 0.23	0.92 ± 0.20
11 years	0.90 ± 0.22	0.89 ± 0.22	0.88 ± 0.23	0.92 ± 0.20
12 years	0.92 ± 0.20	0.91 ± 0.20	0.90 ± 0.21	0.94 ± 0.19
13 years	0.92 ± 0.19	0.93 ± 0.16	0.90 ± 0.19	0.94 ± 0.19
14 years	0.92 ± 0.19	0.94 ± 0.13	0.89 ± 0.20	0.99 ± 0.08
15 years	0.90 ± 0.15	0.89 ± 0.17	0.85 ± 0.18	0.97 ± 0.11
16 years	0.94 ± 0.15	0.96 ± 0.10	0.92 ± 0.17	1.00 ± 0.04
17 years	0.92 ± 0.18	0.97 ± 0.08	0.92 ± 0.18	0.96 ± 0.11
Total	0.92 ± 0.17	0.92 ± 0.18	0.90 ± 0.19	0.95 ± 0.15

VA, visual acuity (Snellen decimal notation); VARE, visual acuity right eye; VALE, visual acuity left eye; VA worst eye, the visual acuity of the eye with the lowest. * Worst eye is the eye with the lowest VA.

**Table 3:** Proportion of children with normal and abnormal visual acuity at each age.

			Abnormal VA		
	Normal VA ^Δ^	Abnormal VA ^α^	Mild Degree *	Moderate Degree **	Severe Degree ***
Age (n (%))					
6 years	259 (91.52%)	24 (8.48%)	23 (8.13%)	1 (0.35%)	0 (0.00%)
7 years	270 (90.60%)	28 (9.40%)	27 (9.06%)	1 (0.34%)	0 (0.00%)
8 years	286 (89.10%)	35 (10.90%)	31 (9.66%)	1 (0.31%)	3 (0.93%)
9 years	268 (83.75%)	52 (16.25%)	41 (12.81%)	5 (1.56%)	6 (1.88%)
10 years	280 (83.33%)	56 (16.67%)	38 (11.31%)	7 (2.08%)	11 (3.27%)
11 years	235 (84.23%)	44 (15.77%)	29 (10.39%)	4 ( 1.43%)	11 (3.94%)
12 years	79 (86.81%)	12 (13.19%)	8 (8.79%)	2 (2.20%)	2 (2.20%)
13 years	54 (90.00%)	6 (10%)	4 (6.67%)	1 (1.67%)	1 (1.67%)
14 years	45 (81.82%)	10 (18.18%)	9 (16.36%)	0 (0.00%)	1 (1.82%)
15 years	32 (76.19%)	10 (23.81%)	10 (23.81%)	0 (0.00%)	0 (0.00%)
16 years	46 (92.00%)	4 (8.00%)	3 (6.00%)	1 (2.00%)	0 (0.00%)
17 years	24 (82.76%)	5 (17.24%)	5 (17.24%)	0 (0.00%)	0 (0.00%)
Total	1878 (86.78%)	286 (13.22%)	228 (10.54%)	23 (1.06%)	35 (1.62%)

VA, visual acuity. Worst eye is defined as the eye with the lowest VA. Δ, normal VA of worst eye (VA ≥ 0.8); α, abnormal VA of worst eye (VA < 0.8); * mild VA deficit of worst eye (0.8 < AV ≥ 0.3); ** moderate VA deficit of worst eye (0.3 < AV ≥ 0.125); *** severe VA deficit of worst eye (VA < 0.125).

## Data Availability

The original contributions presented in this study are included in the article. Further inquiries can be directed to the corresponding author(s).

## Abbreviations

The following abbreviations are used in this manuscript:

ETDRS	Early Treatment Diabetic Retinopathy Study
MAR	Minimum Angle of Resolution
SD	standard deviation
VA	visual acuity
VAR	Visual Acuity Rating
VARE	visual acuity right eye
VALE	visual acuity left eye
WHO	World Health Organization

## Appendix A

**Table A1:** Percentile values 5, 10, 25, 50, 75, 90, and 95 of monocular right eye, left eye and worst eye and binocular visual acuity (Snellen decimal notation) in children aged 6 to 17 years.

	p5	p10	p25	p50	p75	p90	p95
VARE							
6 years	0.7	0.8	1	1	1	1	1
7 years	0.6	0.8	0.9	1	1	1	1
8 years	0.7	0.8	1	1	1	1	1
9 years	0.5	0.65	0.9	1	1	1	1
10 years	0.3	0.7	0.9	1	1	1	1
11 years	0.3	0.6	0.9	1	1	1	1
12 years	0.3	0.8	0.9	1	1	1	1
13 years	0.5	0.7	0.9	1	1	1	1.05
14 years	0.6	0.6	0.9	1	1	1	1.1
15 years	0.6	0.7	0.9	0.95	1	1	1
16 years	0.7	0.8	0.9	1	1	1	1
17 years	0.5	0.5	1	1	1	1	1
VALE							
6 years	0.7	0.8	1	1	1	1	1
7 years	0.6	0.8	0.9	1	1	1	1
8 years	0.6	0.8	0.9	1	1	1	1
9 years	0.5	0.7	0.9	1	1	1	1
10 years	0.2	0.6	0.9	1	1	1	1
11 years	0.2	0.6	0.9	1	1	1	1
12 years	0.5	0.7	0.9	1	1	1	1
13 years	0.65	0.75	0.9	1	1	1	1.05
14 years	0.7	0.8	0.9	1	1	1	1.1
15 years	0.5	0.6	0.9	1	1	1	1
16 years	0.8	0.85	0.9	1	1	1	1
17 years	0.8	0.8	1	1	1	1	1
VA worst eye *							
6 years	0.6	0.8	0.9	1	1	1	1
7 years	0.6	0.8	0.9	1	1	1	1
8 years	0.6	0.7	0.9	1	1	1	1
9 years	0.5	0.6	0.9	1	1	1	1
10 years	0.2	0.6	0.8	1	1	1	1
11 years	0.2	0.5	0.9	1	1	1	1
12 years	0.3	0.7	0.9	1	1	1	1
13 years	0.45	0.7	0.9	1	1	1	1
14 years	0.4	0.6	0.9	1	1	1	1.1
15 years	0.5	0.6	0.8	0.9	1	1	1
16 years	0.5	0.8	0.9	1	1	1	1
17 years	0.5	0.5	1	1	1	1	1
Binocular VA							
6 years	0.8	0.9	1	1	1	1	1
7 years	0.8	0.9	1	1	1	1	1
8 years	0.7	0.9	1	1	1	1	1
9 years	0.55	0.8	1	1	1	1	1
10 years	0.4	0.8	1	1	1	1	1
11 years	0.4	0.7	1	1	1	1	1
12 years	0.5	0.9	1	1	1	1	1
13 years	0.55	0.7	1	1	1	1	1.1
14 years	0.8	0.9	1	1	1	1.1	1.1
15 years	0.8	0.9	1	1	1	1	1
16 years	0.9	1	1	1	1	1	1
17 years	0.7	0.8	1	1	1	1	1

P, percentile; VA, visual acuity (Snellen decimal notation); VARE, visual acuity right eye; VALE, visual acuity left eye; VA worst eye, the visual acuity of the eye with the lowest; ***** worst eye is the eye with the lowest VA.
